# Oncogenomic portals for the visualization and analysis of genome-wide cancer data

**DOI:** 10.18632/oncotarget.6128

**Published:** 2015-10-15

**Authors:** Katarzyna Klonowska, Karol Czubak, Marzena Wojciechowska, Luiza Handschuh, Agnieszka Zmienko, Marek Figlerowicz, Hanna Dams-Kozlowska, Piotr Kozlowski

**Affiliations:** ^1^ European Centre for Bioinformatics and Genomics, Institute of Bioorganic Chemistry, Polish Academy of Sciences, Poznan, Poland; ^2^ Department of Hematology and Bone Marrow Transplantation, Poznan University of Medical Sciences, Poznan, Poland; ^3^ Institute of Computing Sciences, Poznan University of Technology, Poznan, Poland; ^4^ Department of Diagnostics and Cancer Immunology, Greater Poland Cancer Centre, Poznan, Poland; ^5^ Chair of Medical Biotechnology, Poznan University of Medical Sciences, Poznan, Poland

**Keywords:** cBioPortal, COSMIC, IntOGen, PPISURV/MIRUMIR, Tumorscape

## Abstract

Somatically acquired genomic alterations that drive oncogenic cellular processes are of great scientific and clinical interest. Since the initiation of large-scale cancer genomic projects (e.g., the Cancer Genome Project, The Cancer Genome Atlas, and the International Cancer Genome Consortium cancer genome projects), a number of web-based portals have been created to facilitate access to multidimensional oncogenomic data and assist with the interpretation of the data. The portals provide the visualization of small-size mutations, copy number variations, methylation, and gene/protein expression data that can be correlated with the available clinical, epidemiological, and molecular features. Additionally, the portals enable to analyze the gathered data with the use of various user-friendly statistical tools. Herein, we present a highly illustrated review of seven portals, i.e., Tumorscape, UCSC Cancer Genomics Browser, ICGC Data Portal, COSMIC, cBioPortal, IntOGen, and BioProfiling.de. All of the selected portals are user-friendly and can be exploited by scientists from different cancer-associated fields, including those without bioinformatics background. It is expected that the use of the portals will contribute to a better understanding of cancer molecular etiology and will ultimately accelerate the translation of genomic knowledge into clinical practice.

## INTRODUCTION

Cancer encompasses a broad spectrum of diseases (>100) that arise from somatically acquired genetic, epigenetic, transcriptomic, and proteomic alterations that have accumulated in the genomes of cancer cells [1]. These alterations are implicated in hallmark oncogenic cellular processes that are characterized by, e.g., sustained proliferative signaling, resistance to apoptosis, induction of invasion and metastasis, and neoangiogenesis [2]. The somatic loss-of-function or gain-of-function alterations are overrepresented in specific genomic regions, which could indicate their potential suppressive or oncogenic roles, respectively. However, it must be noted that somatic mutations occur on different genetic backgrounds and can sometimes interact with germline mutations, which could modify predisposition to cancer when such mutations occur in cancer-associated genes.

Recent advances in technologies for high-throughput genome analysis, such as microarray-based methods and next-generation sequencing (NGS), have enhanced progress in the field of oncogenomics [3]. These tools were fundamental for the initiation and development of multi-centered cancer genomic projects, such as (i) the Wellcome Trust Sanger Institute's Cancer Genome Project (CGP) [4, 5], (ii) The Cancer Genome Atlas (TCGA) [6–8], and (iii) the International Cancer Genome Consortium (ICGC) cancer genome projects [9, 10]. These projects have been launched for genome-wide analyses of genetic, epigenetic, transcriptomic, and proteomic alterations in hundreds or even thousands of cancer samples. Their general aim is to provide publicly available oncogenomic datasets for the better understanding of the molecular mechanisms that underlie cancer and for the assessment of the influence of specific alterations on clinical phenotypes. Application of the appropriate pipeline for computational interpretation and thought-provoking visualization of the results of oncogenomic projects is crucial to exploring the multidimensional character of genome-wide cancer data [11]. In response to this need, a number of oncogenomic portals were created to assist with accessing the abundant cancer datasets. These portals gather and facilitate the analysis of data with regard to small-size mutation, copy number variation (CNV), methylation, and gene/protein expression. Moreover, they offer a wide range of analysis tools that include the testing of correlations of specific genomic alterations with available clinical information.

Herein, we provide a highly illustrated guide through several web-based oncogenomic portals that were generated to facilitate scientists from different cancer-associated fields, including molecular and clinical oncologists, epidemiologists, and bioinformaticians, with the extraction of meaningful information from expanding oncogenomic sources. Browsing through the portals, prospective users will find a variety of data regarding cancer types and subtypes, oncogenic molecular pathways and cancer-associated genes of interest. All of the portals described below are user-friendly and provide intuitive integration as well as interactive oncogenomic dataset visualizations, and thus, bioinformatics skills and knowledge are not essential to exploring and using these tools. The individual paragraphs listed below present the characteristics and possible utilization of selected web portals. Descriptions and figures that present specific portals were prepared according to their versions from the first half of 2015, and they are summarized in Table 1.

**Table 1:** Main characteristics of the selected oncogenomic portals

database	data source	sites of analysed cancer^1^	organisation of data^2^	oncogenomic data/analyses	link/literature
Tumorscape	Broad Institute	Bd; Bld; Br; Bra; Clr; Eso; GIST; HN; Htp; Kd; Lng; Lvr; Lymph; Msh; Ov; Pnc; Prst; Sk; ST; Stc; Swn; Thr; Utr; also in: cancer cell lines	level i-iii	copy number alterations	http://www.broadinstitute.org/tumorscape/pages/portalHome.jsf; [12]
UCSC Cancer Genomics Browser	TCGA, SU2C Breast Cell Line, Cancer Cell Line Encyclopedia, The Connectivity Map, TARGET, cancer data from literature	Bd; Bld; Br; Bra; Chl; Col; Clr; EG; Eso; HN; Kd; Lng; Lvr; Lymph; Msh; Ov; Pan; Pnc; Prc /Prn; Prst; Rc; Sk; ST; Stc; Thm; Thr; Utr; also: cancer cell lines; cancer data from mouse models	level i-iii	DNA copy number, miRNA/exon/gene/protein expression, DNA methylation, gene-level mutations, PARADIGM pathway activity; clinical, epidemiological, and molecular information	https://genome-cancer.ucsc.edu; [14–18]
ICGC Data Portal	ICGC, TCGA, TARGET	Bd; Bld; Bo; Br; Bra; Clr; Col; Eso; HN; Kd; Lng; Lvr; Lymph; Nb; Ov; Pnc; Prst; Rc; Sk; ST; Stc; Thr; Utr;	level i-iv	simple somatic mutations, copy number somatic alterations, structural somatic mutations, simple germline variants, DNA methylation, gene/protein expression, miRNA expression, exon junction; epidemiological and clinical data	https://dcc.icgc.org; [32]
COSMIC	TCGA, ICGC, cancer data from literature	Bo; Br; EA; Eso; GIST; Htp; Kd; Lvr; Lng; Ov; Pnc; Prst; Sk; Stc; Tst; Thm; Thr; Utr	level iii-iv	somatic mutations, copy number alterations, gene expression	http://www.sanger.ac.uk/genetics/CGP/cosmic; [39–43]
cBioPortal	AMC, BCCRC, BGI, British Columbia, Broad, Broad/Cornell, CCLE, CLCGP, Genentech, ICGC, JHU, Michigan, MKSCC, MKSCC/Broad, NCCS, NUS, PCGP, Pfizer UHK, Riken, Sanger, Singapore, TCGA, TSP, UTokyo, Yale	ACC; Bd; Bld; Br; Bra; Chl; Clr; Eso; HN; Kd; Lng; Lvr; Lymph; MM; Npx; Ov; Pnc; Prst; Sk; ST; Stc; Thr; Utr; also: cancer cell lines	level iii-iv	mutations, putative copy number alterations; mRNA expression, protein/phosphoprotein level; survival analyses	http://www.cbioportal.org; [57, 58]
IntOGen (2014.12)	TCGA, ICGC, cancer data from literature	Bd; Bld; Br; Bra; Clr; Eso; HN; Kd; Lng; Lvr; Lymph; Ov; Pnc; Prst; Sk; Stc; Thr; Utr	level iii-iv	results of the analyses indicating driver alterations and genes; therapies tailored to the mutation profiles of the analyzed patients	http://www.intogen.org/; [67–70]
BioProfiling.de					
PPISURV	for gene expression: Gene Expression Omnibus; for interactome: IntAct, HPRD, Reactome, HumanCyc, NCI_NATURE, PhosphoSitePlus	Bd; Bld; Br; Bra; Col; Htp; Lng; Lvr; Lymph; Ov; Prst; ST; Utr	level iv	survival analyses	http://bioprofiling.de/GEO/PPISURV/ppisurv.html; [81]
MIRUMIR	Gene Expression Omnibus	Br; Eso; Lvr; Lng; Npx; Ov; Prst; Sk	level iv	survival analyses	http://www.bioprofiling.de/GEO/MIRUMIR/mirumir.html; [83]
DRUGSURV	for gene expression: Gene Expression Omnibus; for drugs modulating a gene of interest: DrugBank, Pubchem Bioassay	Bld; Br; Bd; Col; Bra; Lng; Lvr; Lymph; Prst;; ST; Utr	level iv	list of drugs targeting specific genes/cancer types; survival analyses	http://www.bioprofiling.de/GEO/DRUGSURV/index.html; [85]

1 List of abbrieviations of cancer sites. In the brackets there are exemplary cancer subtypes included in the portals.

ACC – adenoid cystic carcinoma; Bd – bladder; Bld – blood; Bo – bone; Br – breast; Bra – brain; Chl – cholangiocarcinoma; Clr – colorectal; Col – colon; EA – eye and adnexa; EG - endocrine glands; Eso – esophagus; GIST – gastrointestinal; HN – head and neck; Htp – hematopoietic; Kd – kidney; Lng – lung; Lvr – liver and biliary tract; Lymph – Lymphoma; Msh – mesothelioma; Mth – mouth; Nb – neuroblastoma; Npx – nasopharynx; Ov – ovary; Pan – pancancer; Pnc – pancreas; Pnx – pharynx; Prc/Prn - pheochromocytoma and paraganglioma; Prst – prostate; Rc – rectum; Sk – skin; ST – soft tissues; Stc – stomach; Swn – schwannoma; Thm – thymus; Thr – thyroid; Tst – testis; Utr – uterine (cerxix and corpus).

2 In oncogenomic portals cancer resources are arranged in different levels of organisation, including: (i) raw, (ii) computationally processed/normalized, (iii) interpreted and (iv) summarized data [3].

### Tumorscape

**Figure 1:**
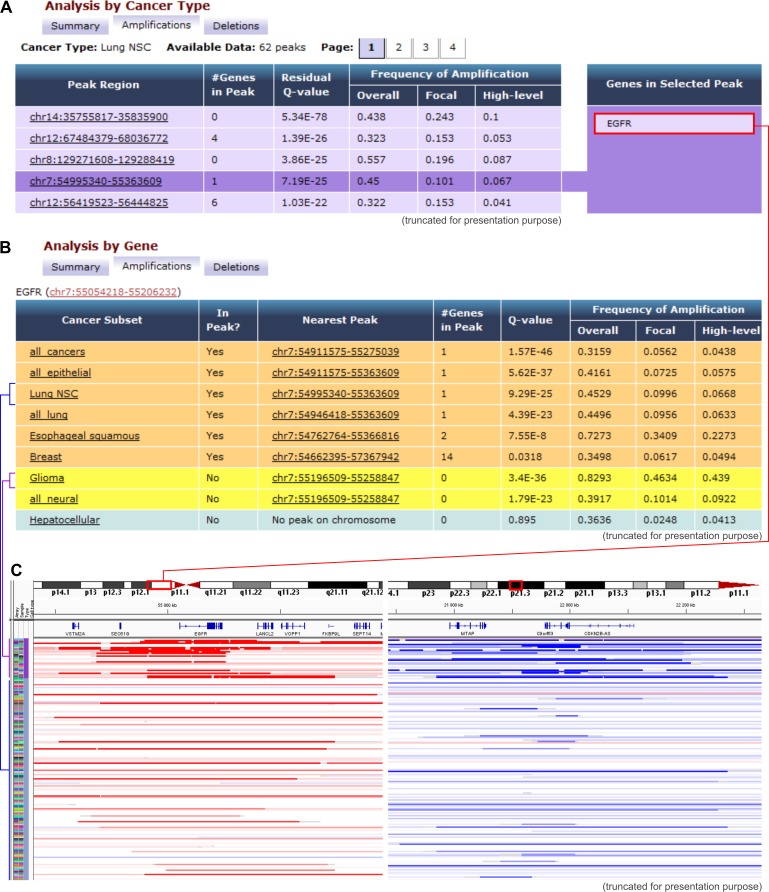
Examples of Tumorscape data analysis and visualization **A.** An example of the results that were obtained with the “cancer-centric” analysis. The table shows a list of genomic regions that were most frequently amplified in lung adenocarcinoma. The q-value represents the likelihood of a random occurrence of the specific amplification/deletion that is calculated based on the background copy number variation. The fourth most frequently amplified region that spans *EGFR* is highlighted. **B.** Results obtained with “gene-centric” analysis; the table depicts a list of cancers in which the representative gene (*EGFR)* is located in or near the frequently amplified region (orange and yellow rows, respectively). **C.** Visualization of chromosomal regions that span the exemplary *EGFR* and *CDKN2A* genes, which are undergoing frequent amplifications and deletions, respectively. The heatmaps show copy number variations of glioma and lung adenocarcinoma samples. Each row represents an individual sample, and red and blue indicate amplification and deletion, respectively.

Tumorscape [12, 13] was developed at The Broad Institute of MIT and Harvard in Cambridge, MA USA. This website was one of the first oncogenomic portals to provide information about cancer copy number changes in a format that was easily accessible to non-bioinformaticians. With this portal, the copy number profiles of over 3,700 cancers (both primary cancers and cell lines) are mapped to the human genome reference sequence and are visualized as heatmap tracks, with the use of the Integrative Genomics Viewer (The Broad Institute). Genomic regions with increased (>2) and decreased (<2) copy number are marked, respectively, in red and blue colors, the intensity of which indicates the amplitude of the copy number changes (Figure 1). The tracks that represent all of the analyzed samples are shown next to one another, forming a panel that allows direct comparison and visualization of all of the analyzed samples. In addition, Tumorscape provides tools that allow “cancer-centric” and “gene-centric” data analyses. The “cancer-centric” analysis (Figure 1A) provides a list of genomic regions that are either significantly amplified or deleted in a specific cancer along with information about the genes that are located in the altered regions. The “gene-centric” (Figure 1B) analysis provides summary statistics of the copy number alterations that affect a gene of interest in a specific cancer type and/or across all cancer types. This summary enables the interpretation of the role of an analyzed gene as a potential oncogene or tumor suppressor.

### UCSC cancer genomics browser

**Figure 2:**
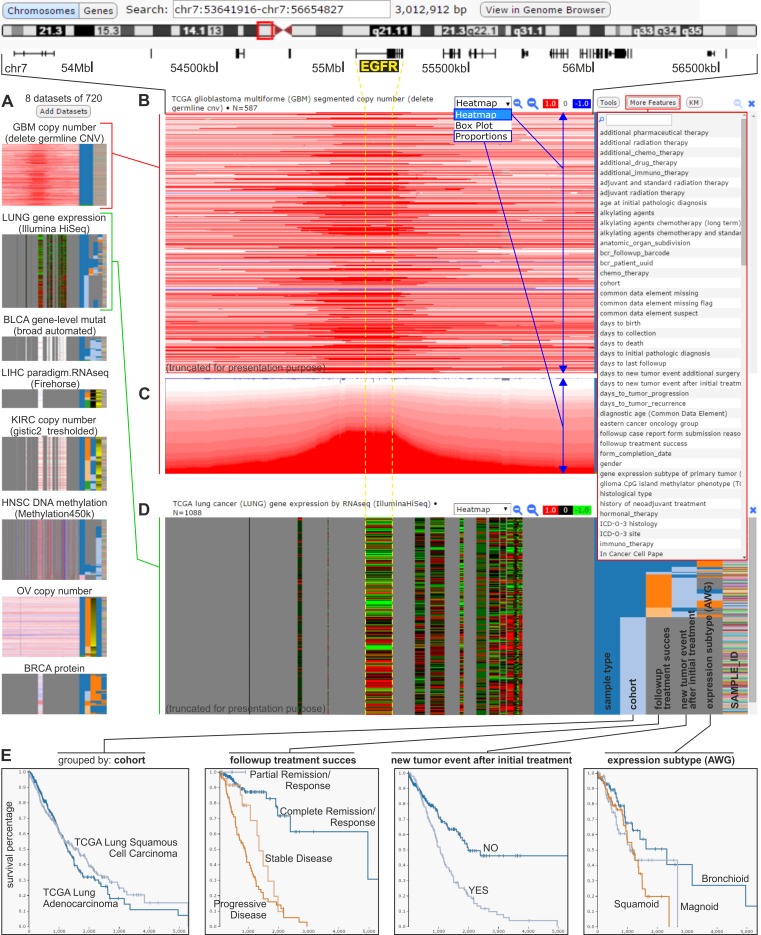
The UCSC Cancer Genomics Browser An example of analysis focused on the *EGFR* genomic region that is conducted concurrently on various oncogenomic data across different cancer types and subtypes. **A.** Small-scale images (icons) of selected datasets that are simultaneously visualized in the browser. Datasets represented by icons are displayed in a column, similar to the datasets from panels B-D. **B.** A heatmap panel that presents the results of the TCGA genome-wide copy number analysis of glioblastoma multiforme (GBM) samples. A screenshot of the GBM dataset was used for presentation, based on the presence of considerable amplification of the genomic region that spans the representative *EGFR*. Each horizontal line (track) represents a specific sample. The red or blue colors indicate, respectively, a gain or loss in the copy number. On the right side of panel B, there is a drop-down list with epidemiological, clinical, and molecular attributes that can be used to sort the presented data (as shown in panel D). **C.** The TCGA copy number data identified in patients with GBM visualized as a proportions plot. **D.** A heatmap panel showing the results of TCGA analysis of gene expression in lung cancer samples in the genes that are indicated above (e.g., *EGFR*). Red and green colors indicate, respectively, upregulation and downregulation of the relative gene expression. The samples are sorted by epidemiological, clinical, and molecular attributes (selected from a drop-down list of attributes), as in panels B and C, shown on the right side of the expression panel. The copy number and expression data presented in panels B-D correspond to the same genomic region indicated above panel B. **E.** Kaplan-Meier plots generated using the attributes of lung cancer samples (shown in the right side of panel D).

The University of California at Santa Cruz (UCSC) Cancer Genomics Browser [14–19] integrates oncogenomic CNV, small-size mutations, methylation, transcriptomic, and proteomic datasets that were obtained in a variety of experiments that were conducted with the use of samples from different cancer types and subtypes. With this portal, all of the oncogenomic information is mapped to the human genome reference sequence and presented as color-coded heatmap tracks. As in Tumorscape, the data from specific experiments are visualized as panels of heatmap tracks in which each track represents an individual sample. Using this portal, the required data can be browsed from the perspective of the whole genome, the exome, a specific chromosome, or a gene. Additionally, there is also the possibility of viewing PARADIGM datasets to gather a sample-specific “gene activity level.” This parameter (obtained using the PARADIGM method) [20] provides the incorporation of pathway interactions (which are deposited in the NCI Pathway Interaction Database) [21] and the integration of data with regard to different types of oncogenomic alterations, e.g., changes in the expression or copy number of a given gene [16]. In the UCSC Cancer Genomics Browser, multiple panels can be simultaneously displayed to visualize different categories of oncogenomic information for a specific cancer type and/or the same category of oncogenomic data for different cancer types (Figure 2A-2D). With this browser, analyses can be concurrently conducted for thousands of samples (oncogenomic datasets) that are sorted by different clinical, epidemiological, and molecular features (Figure 2). These features include survival, histological type, tumor nuclei percent, followup treatment success, new tumor event after initial treatment, neoplasm histologic grade, and tumor necrosis percent, as well as gender, age at initial pathologic diagnosis, tobacco smoking history, cytogenetic abnormalities, and expression subtypes. Apart from the heatmap tracks (Figure 2B), the data presented in specific panels can be summarized and plotted as box-and-whiskers or proportions (Figure 2C).

The datasets can also be statistically processed and depicted with the use of a number of tools, such as the hgSignature, which enables the simultaneous analysis of the expression of several genes, to incorporate an algebraic expression signature as a clinical feature. The inclusion of such a feature to the statistical analysis of cancer data could allow the correlation of the molecular and clinical phenotypes or the subdivision of the clinical phenotypes based on the molecular data [15]. Additionally, a correlation of the available clinical, epidemiological, and molecular features with a patient's survival can be depicted in a Kaplan-Meier plot (Figure 2E). Subgroups of samples (distinguished based on the associated features or genomic signatures) can be compared in terms of the obtained oncogenomic data with the use of various statistical tests [i.e., differences in mean, Wilcoxon, Fisher's exact, Fisher's linear discriminant, Jarque Bera normality, Levene homogeneity of variances (HOV), Brown - Forsythe HOV, and Student's T-tests], which can be adjusted for multiple hypotheses p-values through the Bonferonni and Benjamini-Hochberg false discovery rate (FDR) corrections. Importantly, all of the genomic information that is stored in the UCSC database can be easily downloaded for external analyses.

Successful applications of the UCSC Cancer Genomics Browser in cancer-associated research are described in many papers [22–30]. For example, Wu and colleagues [22] used the statistical tool for the generation of a Kaplan-Meier plot to support the significance of their experimental data. Their study revealed that the up-regulated expression level of *HNF1A-AS1* in lung adenocarcinoma is significantly correlated with the TNM stage, tumor size, and lymph node metastasis. These results are in line with the Kaplan-Meier plot, which indicates that patients with high *HNF1A-AS1* expression overall experienced worse survival compared to patients with low *HNF1A-AS1* expression. The UCSC Cancer Genomics Browser is also broadly used for downloading genomic and clinical data for external analyses [24–26, 30].

It is also noteworthy that the authors of the UCSC Cancer Genomics Browser are currently developing a new oncogenomic platform called UCSC Xena [31], which allows users to upload, visualize, and analyze a custom genomic dataset in the context of the large projects data stored in the web browser. Although the UCSC Cancer Genomics Browser and the UCSC Xena currently coexist, it is anticipated that after adding some vital functionalities, UCSC Xena will replace the UCSC Cancer Genomics Browser [18].

### ICGC data portal

**Figure 3:**
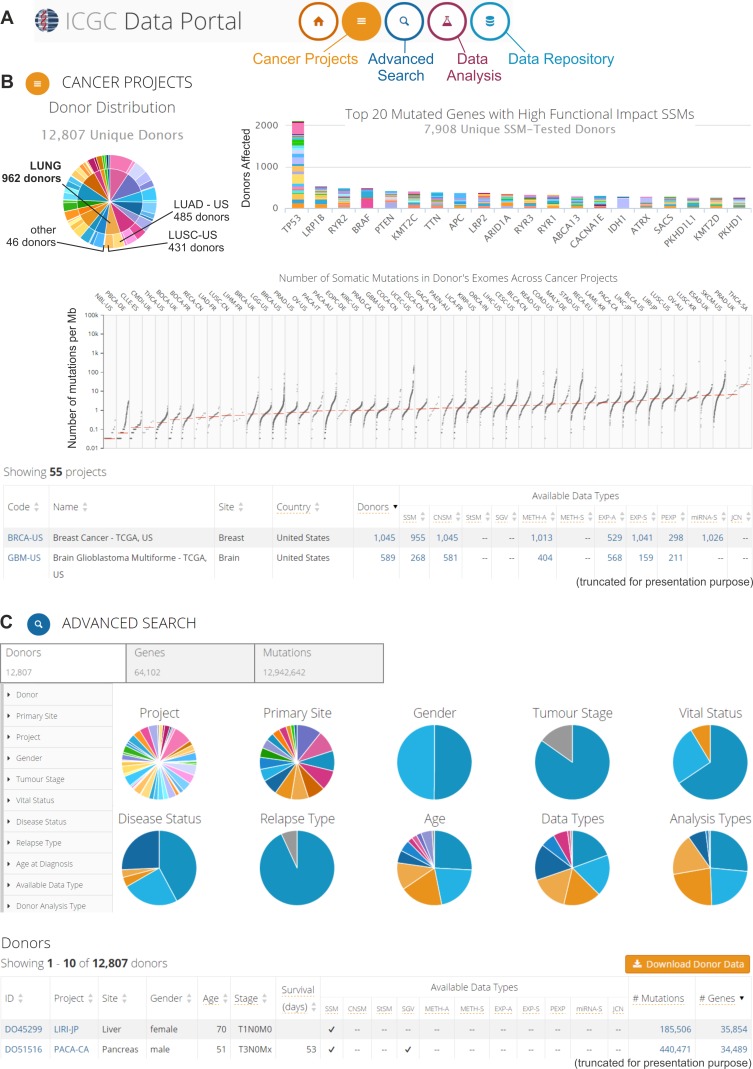
The ICGC Data Portal An example of possible data analyses and visualizations. **A.** Three interactive entry points to the ICGC Data Portal. **B.** The “Cancer Projects” entry point. Screenshot of summary results from all 55 cancer projects. The upper left-hand panel: pie chart that depicts the distribution of cancer types (internal circle) and cancer subtypes/projects (external circle) among the donors, e.g., different lung cancer types and subtypes/projects (indicated in the pie chart). The upper right-hand panel: bar plot that represents the top 20 most frequently mutated genes. Different colors indicate different projects. The middle panel: scatter plot that depicts the distribution of the number of somatic mutations in the donors' exomes across cancer projects. Each dot represents the number of somatic mutations (per 1 Mb) that are identified in the analyzed sample. Vertical lines indicate the median number of mutations. The bottom part of panel B shows a summary of each project. More information about the specific project (types of experimental analyses, available genomic data, most commonly mutated genes, most common mutations, and most affected donors) can be found by clicking at specific project code. **C.** The “Advanced Search” entry point, which enables extended analysis of the oncogenomic data. This screenshot shows the browsing of donor features. The upper left-hand panel depicts features that can be used for filtering the donor data. The middle panel (pie charts) provides a summary of the clinical, epidemiological, and molecular attributes of the donors. The bottom panel represents summary data about specific donors. More information (clinical and genetic) can be found by clicking at the donor ID.

The ICGC Data Portal [32, 33] provides integration and visualization of the results of 55 cancer projects. This portal was created for the analysis of genomic sequence alterations in relation to clinical patient characteristics, such as ethnicity and epidemiological information. With this portal, the oncogenomic data can be analyzed using four interactive entry points: “Cancer Projects,” “Advanced Search,” “Data Analysis” and “Data Repository” (Figure 3A). The “Cancer Projects” (Figure 3B) enables data browsing from distinct projects that focus on the oncogenomic analysis of specific cancer types and subtypes. For each dataset, the provided summary includes a list of available oncogenomic data types, most affected donors, genes most frequently affected by cancer alterations, and most common mutations. It is also possible to use the “keyword search” tool to browse all of the gathered oncogenomic data in terms of a specific gene, mutation, donor, or molecular pathway that is of interest. The integration of external databases, such as the Ensembl [34], OMIM [35], Reactome [36], and COSMIC [37], enables the user to look more broadly at a specific gene, molecular pathway, or mutation in terms of its role in carcinogenesis. The “Advanced Search” (Figure 3C) allows extending the analysis and correlating data with additional clinical (e.g., tumor stage, relapse type, disease status), epidemiological (e.g., gender, age at diagnosis, vital status), molecular (e.g., type of the mutation and its consequence), and technical (e.g., type of sequencing platform used for the analysis) information. The “Data Analysis” entry point allows launching three types of analyses: “Enrichment Analysis,” “Phenotype Comparison,” and “Set Operations.” The “Enrichment Analysis” permits the user to identify groups of gene sets from the selected “universe,” i.e., Reactome Pathways, Gene Ontology (GO) Molecular Function, GO Biological Process or GO Cellular Component, which appear to be statistically significantly over-represented when compared with a custom gene set that is uploaded by the user. The uploaded custom gene set can consist of up to 10,000 genes. The “Enrichment Analysis” is based on a hypergeometric test and Benjamini-Hochberg adjustment for multiple test corrections with the FDR value threshold selected by the user. The “Phenotype Comparison” analysis allows the user to compare some clinical and epidemiological characteristics across patients with various cancer types, whereas the “Set Operations” can be used to distinguish the shared fraction of the analyzed sets, which are depicted in a Venn diagram (e.g., mutations that are causative across several cancer types). “Data Repository” allows all of the ICGC Cancer Project data to be downloaded and analyzed with the use of external programs and tools of interest. An example of ICGC Data Portal utilization for downloading oncogenomic data has already been published [38].

### COSMIC

The Catalogue of Somatic Mutations in Cancer (COSMIC) was developed at the Wellcome Trust Sanger Institute in Hinxton, UK [37, 39–43]. It is the most comprehensive database of somatic mutations in cancer. The portal provides information about the CNV and the expression level of cancer-associated genes that is obtained via the analysis of all of the samples that were tested for specific mutations (both positive and negative results are reported). This tool enables the calculation of the objectivized frequency of mutations in different types of tumors. The records included in COSMIC are derived from two sources: (i) a literature review of over 21,000 research papers and (ii) two projects: TCGA and ICGC. Together, these sources provide information that is obtained from more than a million samples. For almost 20,000 samples, whole-genome sequencing was conducted, which provided complete information about alterations in their genomes. In addition to the above, literature curation allowed the generation of the Cancer Gene Census, which is available under the COSMIC external links; thus far, it is the most reliable list of cancer-associated genes.

**Figure 4:**
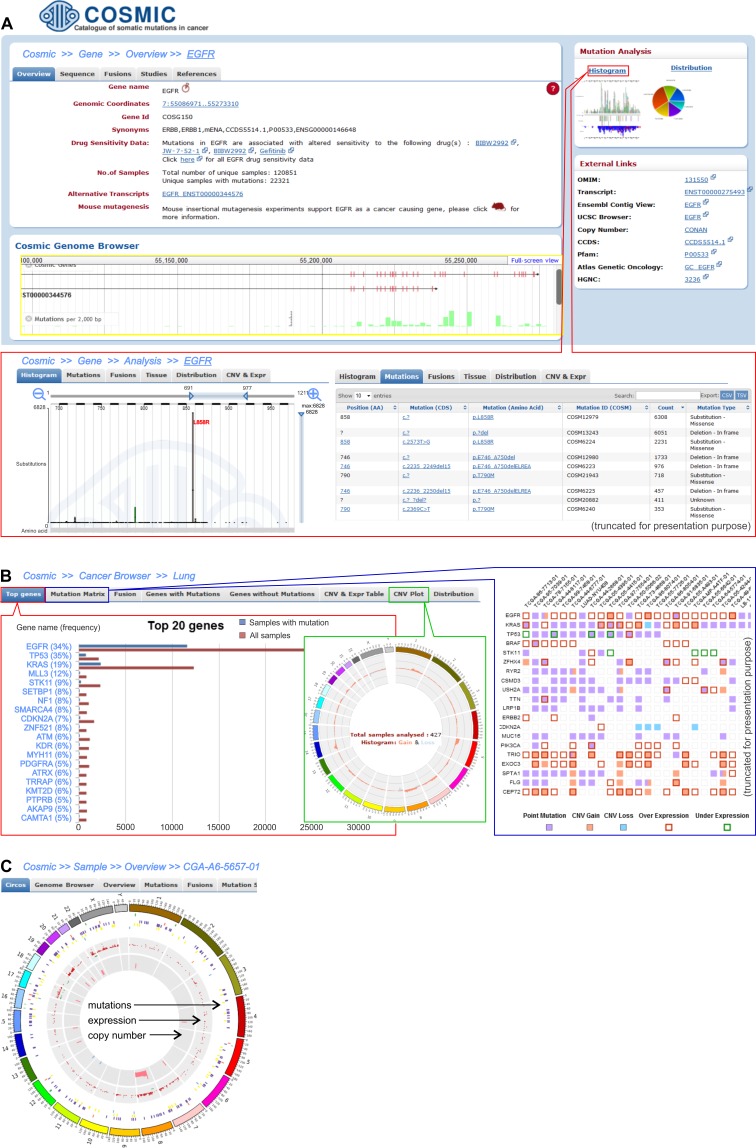
Three levels of data analysis in the COSMIC browser **A.** Screenshot shows exemplary *EGFR* gene data. The upper left-hand panel demonstrates basic information about the gene, whereas the right-hand panel of “Mutation analysis” provides links to the detailed data of mutations that were detected in the *EGFR*. Within the panel, there is a “Histogram” link that allows detailed analysis of the gene alterations, whose features are shown in the framed panel. One of the histograms shows the distribution of *EGFR* tyrosine kinase domain mutations, with the most frequently occurring mutation being L858R. The distribution can also be visualized as a table (on the right). **B.** The screenshots present the results for the representative lung adenocarcinoma cancer type. The left framed panel shows a list of the 20 most frequently mutated genes, whereas the middle and right framed panels display a CNV plot and the Mutation Matrix, respectively. The CNV circular plot shows a summary of the copy number variations across the whole genome of the lung adenocarcinoma. The height of the corresponding bars shows the total number of samples with CNV in a specific region. The Mutation Matrix presents alterations in the most frequently mutated genes (*y*-axis) in the adenocarcinoma samples that have the highest number of alterations (*x*-axis). **C.** Circular plot of all of the alterations (coding mutations, gene expression and CNV) that are detected in an individual exemplary sample (TCGA-A6-5657-01) of adenocarcinoma.

The data integrated in COSMIC can be searched by sample name, by gene name, and via cancer browser (Figure 4). Searching by the sample name allows the user to obtain a genome-wide overview of all of the cancer-associated events (e.g., mutations, gene fusions, and CNV) associated with a sample of interest. The second approach enables the user to overview all of the data that is related to a specific gene, such as its sequence, mutations, fusions, copy number variations, and expression. The data that refer to a specific cancer type (mutations, fusion and copy number and expression alterations of genes) can be retrieved via the cancer browser.

Due to its comprehensiveness, COSMIC is widely used and has been cited in hundreds of publications (e.g., [44–56]). For example, Chen et al. [46] used this database to confirm the presence of specific mutations in the *KRAS*, *NRAS*, and *BRAF* genes in myeloma cell lines. In another study, Ostrow and colleagues [48] took advantage of the Cancer Gene Census to select well-known cancer-associated genes for further analyses of the dynamics of the evolutionary process within tumors, with a focus on breast cancer.

### cBioPortal

The cBioPortal [57–59] was developed at the Memorial Sloan-Kettering Cancer Center in New York City, NY USA. This portal contains genomic data, including copy number alterations, mRNA and microRNA expression, DNA methylation and protein and phosphoprotein abundance, which were obtained for multiple types of cancer. Currently, the portal collects records that were derived from 91 individual cancer studies, in which 31 types of cancer were analyzed with the use of over 21,000 samples. Because the tools that were integrated in the portal perform different types of analyses, different statistical tests can be used to assess the significance in specific analysis (for example, Fisher's exact test can be used to calculate the significance of mutual exclusivity of two genes or the log-rank test can be used to calculate survival analysis significance). All of the portal data can be retrieved in a format that is compatible with the R framework for statistical computing and graphics.

**Figure 5:**
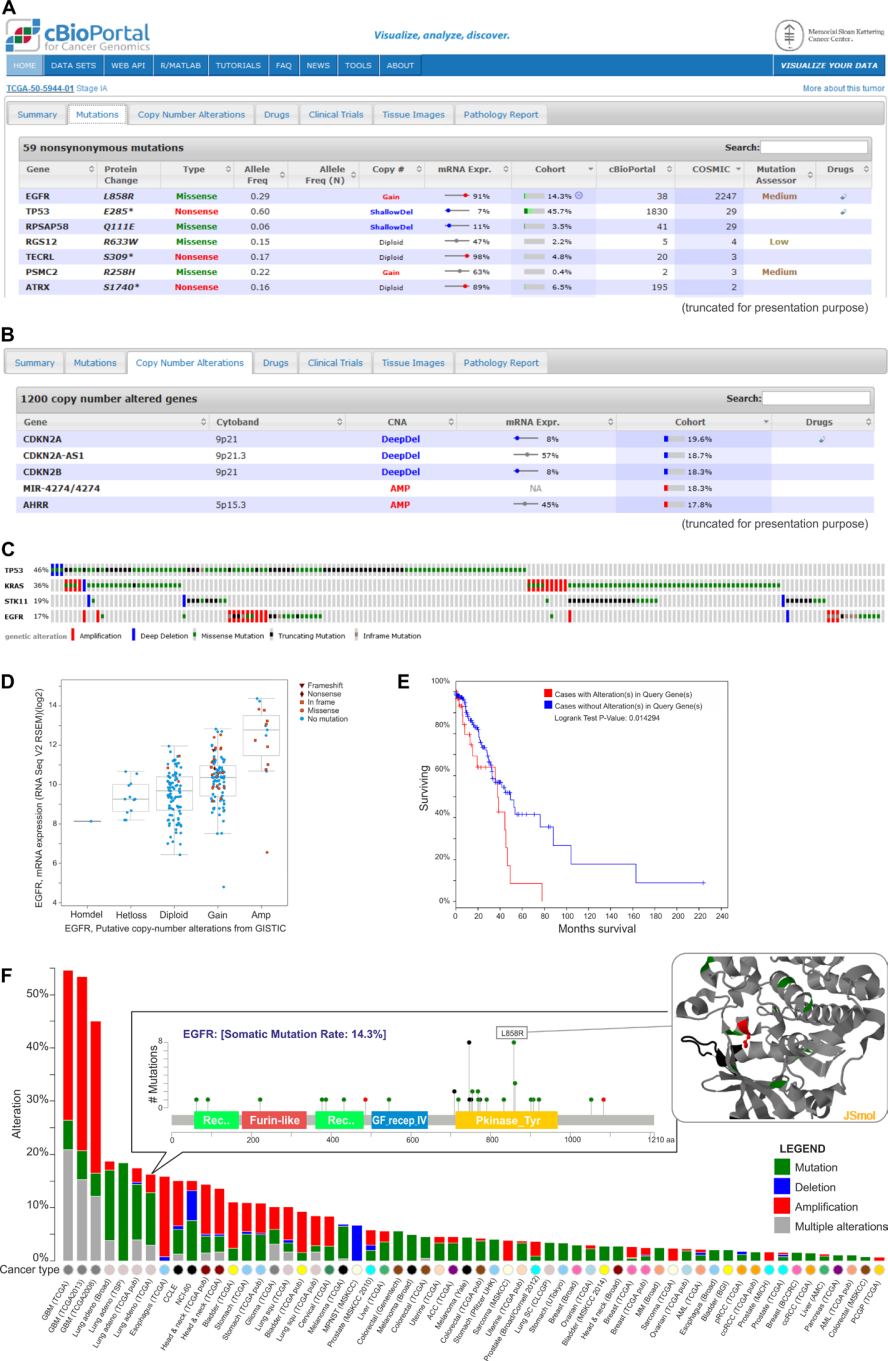
Exemplary data analysis and visualization available in the cBioPortal **A.** The table shows nonsynonymous mutations in the TCGA-50-5944-01 sample of lung adenocarcinoma. They are characterized by the mutation name, its type, its frequency and its effect on the expression of the mutated gene. Additional information on the frequency of specific mutations can be found under the “cBioPortal” and “Cosmic” columns. The table also provides the information about the predictable impact of a given mutation on the gene function (under the Mutation Assessor tool). **B.** Genes with copy number alterations (CNAs) in the TCGA-50-5944-01 sample are shown. The table also contains the information on the frequency of CNA in a specific gene and the effect of the alterations on the gene expression. **C.** Summary of the genomic alterations in four selected genes of lung adenocarcinoma samples. Each column shows an individual tumor sample in which homozygous deletions (blue), amplifications (red), missense mutations (green squares), truncating mutations (black squares) and no mutation changes (grey) were found. **D.** A plot of the correlation between copy number alterations and mRNA expression of the exemplary *EGFR* gene. **E.** Kaplan-Meier plot of overall survival shown for patients with (red) and without (blue) changes in *EGFR.* F. Summary graph of *EGFR* alterations (shown in different colors) in individual studies deposited in the portal. For a selected study, the distribution of the mutations is shown in the inset. For a selected mutation (here L858R), a 3D interactive protein structure can be displayed (the position of the mutation is indicated in red).

Cancer-associated alterations deposited in the cBioPortal can be browsed as (i) the overview of all of the genomic events that were detected in an individual cancer sample (Figure 5A, 5B), (ii) alterations in a specific gene across all of the samples that were included in one study (Figure 5C-5E), and (iii) a comparison of the frequency of the alterations in a given gene across all 91 studies (Figure 5F). For each study, it is also possible to inquire which genes are most frequently altered in the analyzed set of samples. In the cBioPortal, the genomic data are integrated with clinical outcomes, which allows determining whether a specific gene plays a potentially oncogenic role in a given cancer type. Apart from the on-line analysis of data deposited in the portal, there is also the possibility to download the results that were obtained for a specific study. Additionally, the browser enables the visualization of data that is uploaded by the user.

A wide range of tools that are available makes the portal useful in various types of analyses, which has resulted in its popularity and applicability (e.g., [51, 60–65]). For example, the authors of this paper used this portal to determine the correlation between copy number changes and expression level of two miRNA biogenesis genes (*DROSHA* and *DICER1*) that were found to be frequently amplified in lung cancer [63]. Other authors used the cBioPortal for the analysis of the *PARK2* deletion in low-grade glioma and glioblastoma and for the analysis of the correlation between *PARK2* mRNA expression and prognosis in patients [60]. Lu and colleagues used the portal to retrieve copy number data for the design of the model that predicts genetic interactions in human cancer [61].

### IntOGen

The Integrative Oncogenomics Cancer Browser (IntOGen) [66] was developed by the Biomedical Genomics Group integrated in the Research Unit on Biomedical Informatics of the University Pompeu Fabra, Biomedical Research Park in Barcelona. The browser contains the results of computational secondary analyses of oncogenomic data from several large genome-wide projects. The analyses were focused on the selection of cancer-associated genes that are known as drivers. IntOGen is one of the most dynamically developing and updating oncogenomic browsers.

In the initial release of the browser, catalogued cancer data were provided in a set of three integrated web-based sub-portals, namely, the IntOGen Arrays [67], IntOGen TCGA [68], and IntOGen Mutations [69], which allowed the browsing of visualized cancer data from different perspectives. The first sub-portal, i.e., the IntOGen Arrays, exploited cancer data on genome-wide expression and copy number for analyses aimed at selecting genes and molecular pathways that are associated with specific cancer types and subtypes [67]. Analyses provided by the other two IntOGen sub-portals were performed on a partially different set of oncogenomic data but with the use of a similar rationale. In the IntOGen TCGA, the set of somatic sequence alterations identified by exome sequencing of over 3,000 tumors from 12 cancer types (TCGA pan cancer data) was used for analyses focused on the identification of cancer-associated genes, i.e., drivers [68]. The IntOGen Mutations was focused on the evaluation of the role of somatic sequence variants in carcinogenesis and the identification of cancer drivers. In addition to the TCGA data, this sub-portal took advantage of the results from other large projects, e.g., the ICGC. The portal provided results obtained via the analyses of over 4,500 cancer exomes/genomes from 13 cancer types [69]. The results previously gathered in the interactive web-based platforms are currently available in the form of downloadable databases at the IntOGen site [66].

The introduction of a new release of IntOGen (release 2014.12) was aimed at building a bridge between molecular oncogenomics and clinical practice (the personalization of medicine) [70]. Nuria Lopez-Bigas and other co-authors of the browser proposed a strategy of “*in silico* prescription” of tailored anticancer therapy. In the first stage of the strategy, a secondary computational analysis of oncogenomic data from 6,792 patients of 28 different cancer types was performed. The analysis was focused on the evaluation of the role of somatic sequence alterations (including simple somatic variants, copy number alterations and fusion events) in carcinogenesis and the identification of cancer drivers. The drivers were selected when focusing on the following factors: mutation frequency in comparison to background (MutSigCV tool [47]), the presence of highly functional mutations (Oncodrive FM tool [71]), and regional clustering of mutations (Oncodrive CLUST tool [72]) [68, 70]. Although, all of the above tools take advantage of various algorithms and statistical methods, they all are based on similar principles and utilize similar oncogenic gene features. It is important to note that all of the implemented algorithms are supported by appropriate statistical tests. Information about the 459 identified driver genes, including their “mode of action” [loss-of-function (LoF), gain-of-function (GoF) or switch-of-function (SoF)] as assessed with the use of the OncodriveROLE tool [73], is deposited in the Cancer Drivers Database. It can be either interactively visualized in the IntOGen web site [66] or downloaded for external analysis. In further stages of the strategy, Rubio-Perez and colleagues created the Cancer Drivers Actionability Database, which catalogues the already available and candidate therapies (under preliminary research or clinical trials) that are tailored to the cancer genomes of patients who were analyzed in the first stage. The Cancer Drivers Actionability Database can also be downloaded from the IntOGen website [66]. Additionally, the IntOGen portal can be exploited for the analysis of external data in the context of a single tumor or a cohort of tumors.

IntOGen is increasingly used by scientists from various cancer-associated fields for confirmation or identification of a potential driver role of genes of interest (e.g., selected based on experimental results) [74–78]. For example, Kovac and colleagues used IntOGen and MutSigCV programs for computational validation of 20 candidate papillary renal cell carcinoma (pRCC)-specific driver genes, which were selected based on the sequencing analysis of 31 exomes or genomes of pRCCs. The computational analysis of TCGA pRCC data for somatic single nucleotide variants (SNVs) in the candidate genes revealed significantly mutated genes and confirmed *SETD2, BAP1, NFE2L2* and *CUL3* as drivers, with a more modest degree of support for some other genes from a set of experimentally predefined candidates [74].

### BioProfiling.de portal

**Figure 6:**
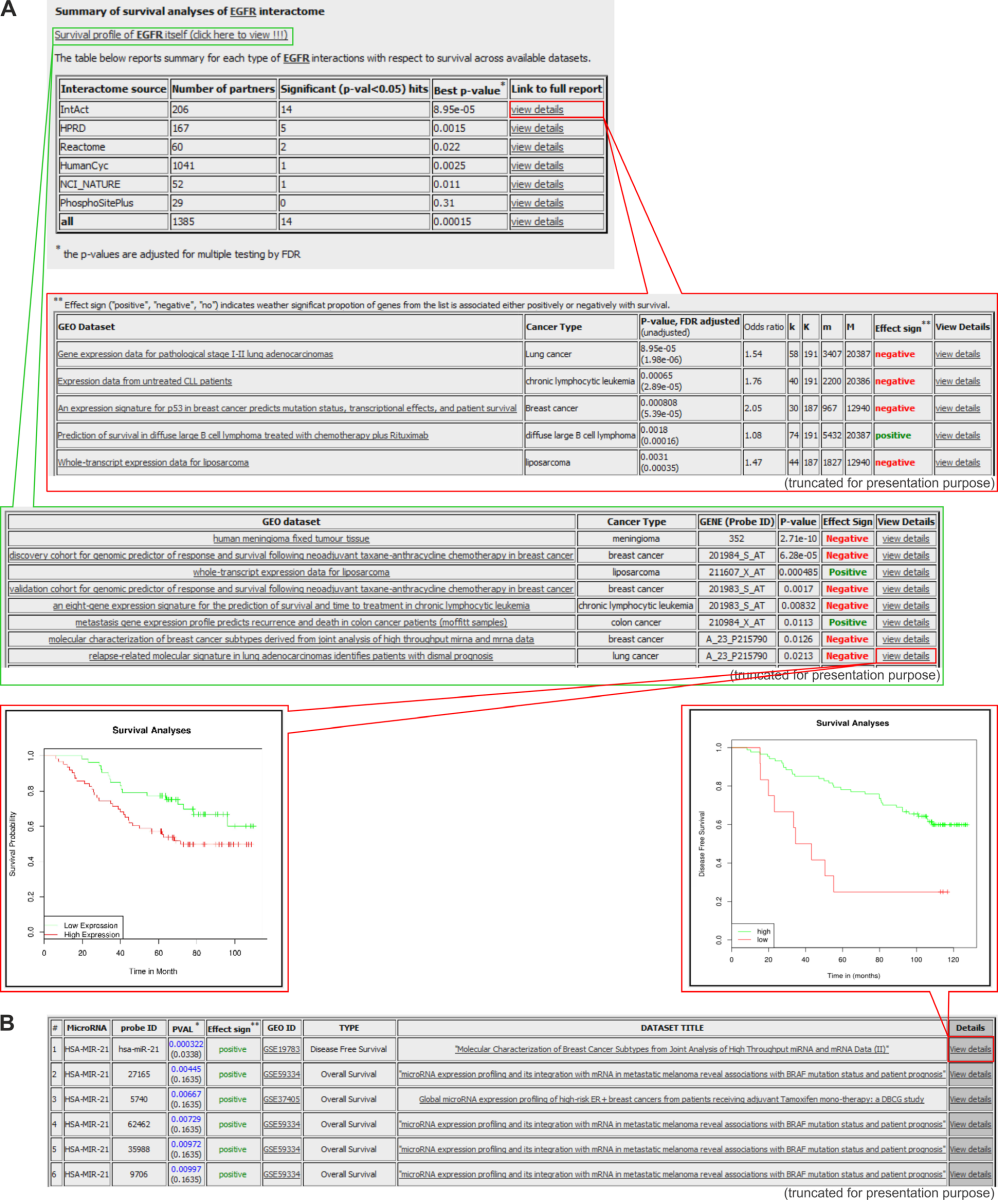
Exemplary results generated in the PPISURV and MIRUMIR databases **A.** Results generated with the PPISURV. Survival analysis shown for representative *EGFR* and its interactome. From the top: the first table depicts the summary of *EGFR* interactions that are annotated according to different interactomes across the available datasets. The last column of the table provides a link for more detailed characteristics of a selected interactome (shown in the second table). It includes the results of the analysis of the influence of the particular interactome on survival determined for all of the available datasets. The third table presents datasets on the direct correlation between *EGFR* expression and survival. The last column of the table is a link for the visualization of the data in the Kaplan-Meier graph. The exemplary graph shows the influence of *EGFR* expression on survival in lung adenocarcinoma patients. **B.** Results generated with MIRUMIR. The table shows a summary analysis for a representative microRNA-21 on the influence of its expression on survival in a specific cancer type. The inset represents the Kaplan-Meier graph of the effect of the microRNA expression on disease-free survival in breast cancer.

The BioProfiling.de portal [79, 80] contains three distinct databases: PPISURV [81, 82], MIRUMIR [83, 84], and DRUGSURV [85, 86]. The main purpose of PPISURV [81, 82] is the identification of important cancer-associated genes that do not have direct impact on the cancer survival outcome but nevertheless affect cancer by various interactions with other genes. Such a map of connections is called a “gene interactome”; it is created based on several external databases, which deposit information about the following: direct protein interactions (deposited in the IntAct Molecular Interaction Database [87]), regulatory and signaling pathways (Reactome, NCI Pathway Interaction Database, and HumanCyc databases) [21, 36, 88], and protein post-translational modifications (PhosphoSitePlus database) [89]. PPISURV allows users to analyze the influence of the gene interactome as well as a gene of interest on survival (Figure 6A). These analyses are performed with the use of over 40 whole transcriptome expression studies that were performed with the use of approximately 8,000 samples that represent 17 types of cancer.

The MIRUMIR provides a similar type of analysis as the PPISURV; however, it is focused on the impact of specific microRNA gene expression on survival in specific cancer types. Either MIRUMIR or PPISURV enable the visualization of survival data via Kaplan-Meier graphs, showing the influence of the expression of a gene of interest on survival in a specific cancer type (Figure 6B).

The third database that is incorporated in the BioProfiling.de portal is DRUGSURV [85]. DRUGSURV provides the opportunity to explore the survival effect of expression alterations of genes that are known to be modulated by a selected drug. This database includes information about approximately 1,700 drugs that were approved by the Food and Drug Administration (FDA), along with approximately 5,000 experimental drugs. A specific drug, cancer type or gene can be queried and investigated in terms of its anticancer potential.

The advantage of the tools that are available in the BioProfiling.de portal is that all of them provide results that are supported by appropriate statistical analysis (the R statistical package), which is not always available for the tools in the other oncogenomic portals. A false discovery rate control procedure is implemented to adjust the p-values when there is multiple testing.

The usefulness of the above-mentioned databases has been confirmed in a number of publications (e.g., [63, 90–98]). For example, Schittek et al., [90] used the PPISURV to perform survival analysis on patients who were stratified based on the expression of *CK1* gene isoforms (*CSNK1A1*, *CSNK1D*, and *CSNK1E*) in different cancers. In another study [99], MIRUMIR was used to evaluate the potential of miR-200c and miR-141 to serve as biomarkers in breast cancer.

## CONCLUSIONS

Since the initiation of large-scale oncogenomic projects, a variety of databases and web-based portals have been created to enable the interactive visualization and interpretation of the abundant genome-wide cancer data. The range of available web-based portals is not limited to those described in our review. Among other noteworthy portals that provide sets of visualization tools that are helpful for oncogenomic data analysis are Oasis [100, 101], Oncomine [102, 103], Cancer Genetics Web [104], and CaSNP [105, 106]. In short, Oasis is a recently launched open-access web portal for explanatory analysis of cancer data. This portal was developed based on a custom version of the BioMart framework that was designed for oncogenomics data analysis, and it provides a unique set of visualization tools. Oncomine is another portal that provides useful visualization and analytical tools, which can browse and analyze over 715 expression and sequence alteration datasets. The Cancer Genetics Web is a web-based tool that can be used to gather literature that is related to a specific cancer type/predisposing syndrome or a gene of interest that is potentially associated with cancer. This tool provides a short summary about a disease and gene of interest, as well as a list of the latest publications and useful external links. Another interesting feature of the Cancer Genetics Web portal is a colorful panel of summarizing keywords that are available for each gene. The fourth portal is CaSNP, which gathers the results of genome-wide CNA profiling that was performed with the use of SNP arrays across 34 different cancer types. In most of the portals, the datasets and methods that are applied in their analyses and graphical presentations are continually updated. As a result, the portals deliver complex pictures of cancer genome alterations and their potential impact on cancer molecular pathogenesis. Importantly, the portals are very intuitive and address a wide community of researchers, who are not necessarily familiar with advanced computational methods. The users can take advantage of oncogenomic portals to further explore the cancer molecular basis and select new candidate cancer-associated genes for experimental validation. Regardless of current interest in exploring data that is gathered in the portals, the usefulness of the tools that are available in the oncogenomic portals will be verified in time by the users. Ultimately, it is expected that the utilization of the portals for the analysis of expanding oncogenomic data will make a substantial contribution to our understanding of cancer molecular etiology and the translation of extended cancer genomic knowledge into clinical practice.
